# Progression of diabetic nephropathy and adverse renal outcomes: possible involvement of Toll-like receptor 4 expression

**DOI:** 10.1007/s10157-026-02849-2

**Published:** 2026-03-24

**Authors:** Ayano Saito, Fumito Abe, Masaya Saito, Mako Hashimoto, Tatsuro Kanazawa, Takuya Kumagai, Mai Sakaguchi, Futaba Ishii, Takahiro Nakayama, Hideki Wakui, Naoto Takahashi

**Affiliations:** 1 Department of Hematology, Nephrology, and Rheumatology, Akita University Graduate School of Medicine, Akita, Japan; 2 Department of Nephrology and Rheumatology, Nihonkai General Hospital, Yamagata, Japan

**Keywords:** Chronic kidney disease, Diabetic nephropathy, Interstitial fibrosis and tubular atrophy, Renal outcome, Toll-like receptor

## Abstract

**Background:**

Diabetic nephropathy (DN) remains a leading cause of end-stage kidney disease despite current therapies. Experimental data implicate Toll-like receptor 4 (TLR4) in DN pathogenesis; however, human evidence, particularly on histological severity and long-term outcomes, is limited. We hypothesized that renal TLR4 expression is correlated with tissue injury and adverse prognosis in DN.

**Methods:**

In 146 adults with biopsy-confirmed DN, we evaluated TLR4 expression in the glomeruli and proximal tubules using immunohistochemical staining. Histological injuries were scored according to the Renal Pathology Society classification. TLR4 expression was graded in the proximal tubular and glomerular epithelial cells. We subsequently investigated whether TLR4 expression is associated with kidney histological damage and whether it relates to renal prognosis.

**Results:**

There was a significant difference in the severity of glomerular lesions across different levels of glomerular epithelial TLR4 expression. Additionally, the extent of interstitial fibrosis and tubular atrophy (IFTA) and interstitial inflammation significantly differed across different levels of proximal tubular TLR4 expression. Overall, TLR4 status did not predict kidney failure-free survival; however, among patients with IFTA < 50% (n = 63), moderate-to-severe tubular TLR4 expression was associated with worse survival than negative-to-mild expression (*p* = 0.021). In the low-IFTA subgroup, there were significant differences in systolic blood pressure, proteinuria, and total cholesterol levels across the levels of tubular TLR4 expression.

**Conclusion:**

These findings indicate possible involvement of TLR4 expression in histological injury and poor renal prognosis in DN, especially before the development of extensive fibrosis. TLR4 and its endogenous ligands are potential novel therapeutic targets for DN.

**Supplementary Information:**

The online version contains supplementary material available at 10.1007/s10157-026-02849-2.

## Introduction

Diabetic nephropathy (DN) is a significant cause of chronic kidney disease and end-stage kidney disease and occurs in approximately 40% of patients with type 2 diabetes [1]. Despite current therapeutic strategies, the risk of DN onset and progression remains substantial. Hyperglycaemia and hypertension associated with diabetes lead to haemodynamic and metabolic abnormalities. These mechanisms are believed to underlie the onset and progression of DN partly by driving glomerular hyperfiltration, inflammation, and fibrosis [2].

Inflammatory processes in DN involve various cytokines and chemokines that contribute to disease onset and progression [3, 4]. Over the past decade, Toll-like receptor (TLR)-mediated mechanisms have been recognised as critical contributors to this process [5–9]. TLRs are essential for the innate immune system, comprising the foundational immune response in all living organisms. Innate immunity recognises pathogen-associated molecular patterns (PAMPs) in bacteria and viruses, enabling a rapid response to foreign substances [10]. TLRs recognise PAMPs and detect endogenous ligands, such as damage-associated molecular pattern molecules, highlighting their involvement in autoimmune diseases and metabolic syndromes [11, 12]. TLRs are present in immune cells, such as macrophages, lymphocytes, and neutrophils, and in various other cell types, including epithelial and endothelial cells and fibroblasts. Ten types of TLRs, each containing a corresponding ligand, have been identified in humans [13]. For instance, TLR2 recognises ligands, such as lipoproteins, peptidoglycan, and heat shock protein (HSP) 70, whereas TLR4 recognises lipopolysaccharides, such as high mobility group box (HMGB) 1, HSP60, and HSP70 [13]. TLR activation is initiated by ligand binding, which brings the cytoplasmic Toll/interleukin-1 receptor domains into proximity, leading to the recruitment of adaptor molecules, such as myeloid differentiation primary response 88 (MyD88). MyD88 is involved in the downstream signalling of all TLRs, except TLR3, activating nuclear factor kappa-light-chain enhancer of activated B cells (NF-κB) and inducing pro-inflammatory cytokines, thereby promoting inflammation [10].

Reports linking DN and TLRs date back to 2010, when Li et al. demonstrated increased renal TLR2 expression in diabetic rats [14]. In that study, TLR2 upregulation in renal tissue was accompanied by increased MyD88 and monocyte chemoattractant protein 1 expressions, activation of NF-κB, and enhanced macrophage infiltration, providing the first direct evidence in the kidney that TLR signalling contributes to inflammation in DN. Subsequently, TLR2 and TLR4 are highly expressed in human renal tubular epithelial cells under hyperglycaemic conditions [7]. Although additional studies have consistently demonstrated an association between TLR2 and TLR4 signalling and DN, investigations of TLRs in human DN remain scarce. Consequently, the precise extent to which TLR overexpression contributes to renal tissue injury and affects the renal prognosis remains unknown. We hypothesised that TLR4 expression correlates with the severity of renal histopathological injury in human DN and, ultimately, with renal outcomes.

## Materials and methods

### Study design and setting

We conducted a single-centre, retrospective cohort study of biopsy-proven DN using medical chart review, immunohistochemical assessment of kidney biopsy specimens, and longitudinal follow-up to evaluate renal outcomes at Akita University Hospital and its affiliated hospitals. This study was approved by the Ethical Review Committee of Akita University Hospital (approval number: 2945). The current study complied with the tenets of the Declaration of Helsinki and the Ethical Guidelines for Medical and Health Research Involving Human Subjects. Study details were posted on our institutional website, and an opt-out mechanism allowed patients to request the exclusion of their data from research use. This report complies with the consensus-based standards of the Strengthening the Reporting of Observational Studies in Epidemiology Statement [15].

### Participants

**Fig. 1:**
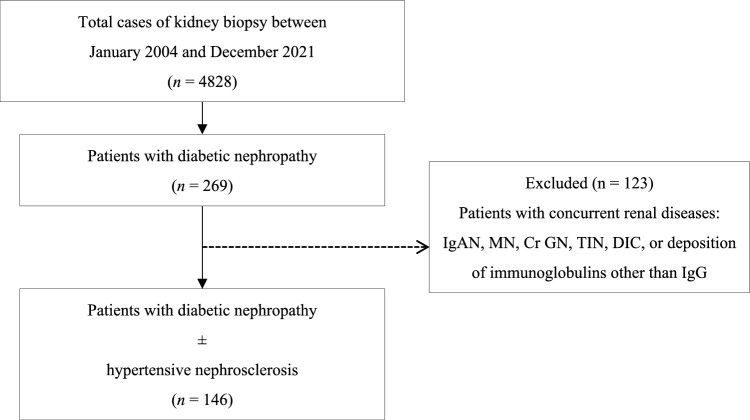
Cohort selection and the inclusion and exclusion criteria used in this study. Abbreviations: IgAN, IgA nephropathy; MN, membranous nephropathy; Cr GN, crescentic glomerulonephritis; TIN, tubulointerstitial nephritis; DIC, disseminated intravascular coagulation

We retrospectively enrolled 146 patients with biopsy-proven DN who underwent kidney biopsy at our institution and its affiliated centres between January 2004 and December 2021. Patients with concomitant renal diseases, including immunoglobulin A and membranous nephropathy, were excluded. Given that many patients with DN also exhibit hypertensive nephrosclerotic changes, our study population included DN cases with concurrent hypertensive nephrosclerosis. Figure 1 shows the cohort selection process employed in this study.

### Measurement

#### Patient characteristics

At the time of biopsy, the following baseline characteristics were recorded: age; sex; body mass index (BMI); blood pressure (BP); use of antihypertensive drugs; estimated glomerular filtration rate (eGFR); glycated haemoglobin (HbA1c), serum albumin, total cholesterol, and C-reactive protein (CRP) levels; degree of proteinuria and haematuria; and presence of diabetic retinopathy. In Japan, the measurement method for HbA1c changed from the Japan Diabetes Society method to the National Glycohemoglobin Standardization Program in April 2012. We added 0.4% to the HbA1c values measured before April 2012.

#### Kidney histopathology

Kidney biopsy specimens were evaluated independently by two nephrologists. For each sample, a minimum of five glomeruli were examined. The severity of interstitial fibrosis and tubular atrophy (IFTA) was assessed through visual estimation. We evaluated renal lesions using the Renal Pathology Society (RPS) classification of DN [16].

#### Immunohistochemistry for Toll-like receptor 4

Formalin-fixed, paraffin-embedded kidney biopsy specimens were sectioned at 4 µm and processed for immunohistochemical staining of TLR4. Following deparaffinisation, antigen retrieval was performed using microwave irradiation for 15 min in 10 mmol/L citrate buffer (pH 6.0). Sections were then blocked, permeabilised with 0.5% Triton X-100, re-blocked, and incubated 60 min at 37 °C with a rabbit anti-TLR4 antibody (Abcam, ab22048) diluted 1:100 in Can Get Signal® Solution A (Toyobo, NkB-501). A polymer-based secondary antibody (HISTOFINE Simple Stain MAX-PO MULTI, Nichirei, 424,151) was used. Visualisation was achieved using ImmPACT™ DAB (Vector Laboratories, SK-4105), and nuclei were counterstained with Mayer’s haematoxylin. For histological assessment, a minimum of five glomeruli per patient were evaluated. Podocyte TLR4 immunostaining was assessed using a previously reported semiquantitative 0–3 scoring system [17]. TLR4-positive podocytes were present, staining was consistently observed in a diffuse pattern, and a “focal” distribution (i.e., < 50% positive glomeruli) was not observed. Therefore, for the primary analyses, podocyte TLR4 staining was categorized as negative or positive to enhance reproducibility and ensure robust interobserver agreement. For the semiquantitative assessment of tubular epithelial TLR4 expression, staining was categorized as negative (< 10%), mild (10% to < 40%), or moderate-to-severe (≥ 40%), based on a grading system described in a previous report with slight modifications [18]. Staining evaluation and scoring were performed in a blinded manner by two independent observers, and discrepancies were resolved by consensus review.

We also attempted immunohistochemical staining for TLR2 in parallel with TLR4; however, TLR2 exhibited positive staining for TLR2 was observed in the control samples (TBMD), indicating non-specific background staining under our experimental conditions. Due to the limited interpretability of the TLR2 signal, subsequent analyses focused exclusively on TLR4, for which the staining performance was acceptable and reproducible.

### Statistical analyses

For baseline characteristics, continuous variables are reported as medians (interquartile ranges), whereas categorical variables are presented as numbers and proportions.

#### Primary analysis

We evaluated significant differences in the histological severity of DN across different TLR4 expression levels using the Mann–Whitney U test for glomerular injuries and the Kruskal–Wallis test for tubular injuries. To evaluate the independent relationships between TLR4 expression and pathological variables, we performed multivariable logistic regression analyses. Variables of pathological relevance were entered into the multivariable model. Results are presented as adjusted odds ratios (ORs) with 95% confidence intervals (CIs). A two-sided p value < 0.05 was considered statistically significant.

#### Secondary analyses

We analysed the association between TLR4 expression and renal prognosis. The observation period was extended from the date of the kidney biopsy to 31 May 2022. Kidney failure-free survival was evaluated using a composite endpoint of kidney failure**—**defined as the initiation of renal replacement therapy, death attributable to renal failure—and nonrenal death. Kidney failure-free survival was evaluated using Kaplan–Meier curves and compared using the log-rank test. Existing evidence links renal TLR4 expression to subsequent kidney function decline in early-stage DN [8]. IFTA is an established independent predictor of renal outcomes in DN [19], and an IFTA score of 3 (≥ 50% involvement) in the RPS classification is the most severe prognostic category. In this context, we dichotomized IFTA at 50% (< 50% vs. ≥ 50%) for our analyses. All analyses were performed using IBM SPSS Statistics for Windows version 28.0 (IBM Corp., Armonk, New York). *p* values < 0.05 were considered statistically significant in all analyses.

Missing data were handled as follows: analyses were performed on an available-case basis; observations with missing values for a given variable were excluded list-wise from that specific analysis but were retained for analyses in which their data were complete. No imputations were performed.

## Results

### Patient characteristics in diabetic kidney disease

**Fig. 2:**
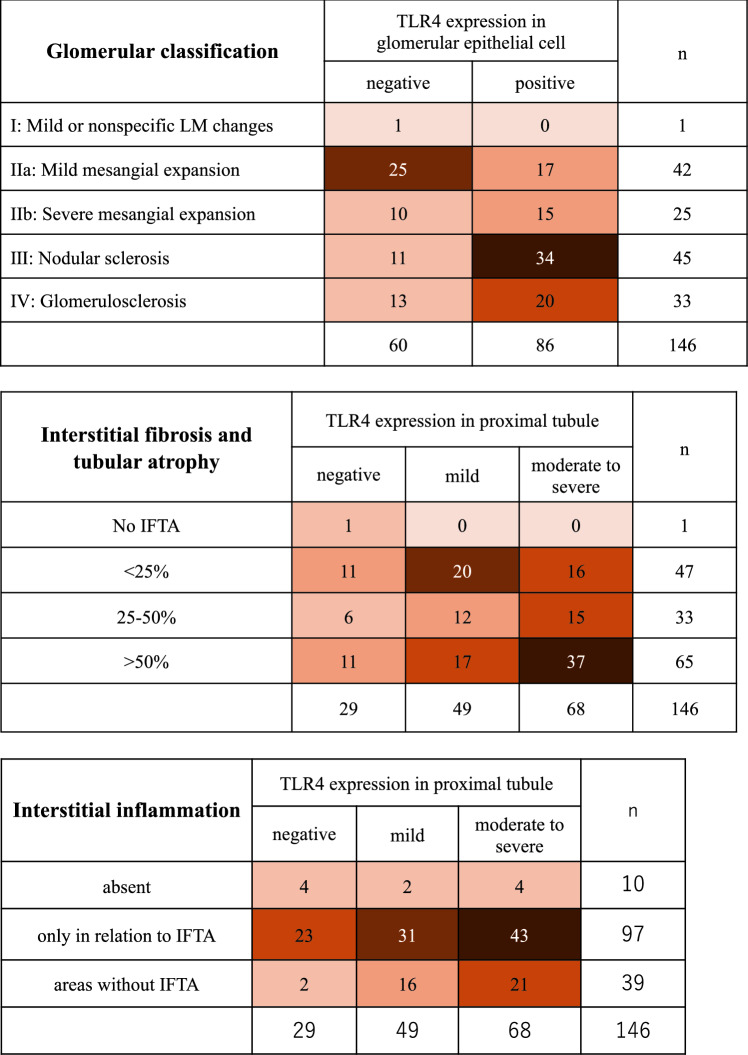
TLR4 expression and the histological features of DN. Abbreviations: TLR, Toll-like receptor; DN, diabetic nephropathy; IFTA, interstitial fibrosis and tubular atrophy; LM, light microscopy

The median patient age was 61 years, and most patients were male (79%). The median eGFR and urinary protein excretion were 38 mL/min/1.73 m^2^ and 4.2 g/gCr, respectively, indicating that most patients were in the overt diabetic nephropathy stage (Table 1). Glomerular lesions were the most frequent RPS classes IIa and III, accounting for 29% and 31% of cases, respectively (Fig. 2). IFTA involving > 50% of the cortical area was present in 45% of the patients, and interstitial inflammatory cell infiltrates confined to areas of IFTA (‘only in relation to IFTA’) were observed in 66% of the patients (Fig. 2). With respect to vascular lesions, most specimens exhibited severe arteriolar hyalinosis and arteriosclerosis (Supplementary Fig. 1).

**Table 1:** Characteristics of patients with DN

	n = 146
Sex, n, male/female, n = 146	109/37
Age, years, median, [IQR], n = 146	61 (49–69)
BMI, kg/m^2^, median, [IQR], n = 136	25.3 (23.2–28.7)
Systolic BP, mmHg, median, [IQR], n = 144	148 (131–161)
Diastolic BP, mmHg, median, [IQR], n = 144	80 (71–89)
Antihypertensive drugs, n, (%), n = 146	123 (84)
eGFR, mL/min/1.73 m^2^, median, [IQR], n = 146	38.1 (25.2–52.8)
HbA1c, %, median, [IQR], n = 146	6.5 (6.0–7.4)
Serum albumin, mg/dL, median, [IQR], n = 146	3.0 (2.4–3.7)
Total cholesterol, mg/dL, median, [IQR], n = 136	202 (171–237)
CRP, mg/dL, median, [IQR], n = 143	0.10 (0.04–0.20)
Proteinuria, g/gCr, median, [IQR], n = 113	4.2 (2.8–9.6)
Proteinuria, g/day, median, [IQR], n = 111	4.0 (2.1–7.2)
Haematuria (RBC ≥ 5/HPF), n, (%), n = 146	64 (44)
Diabetic retinopathy, n, (%), n = 118	68 (58)

DN, diabetic nephropathy; BMI, body mass index; BP, blood pressure; eGFR, estimated glomerular filtration rate; CRP, C-reactive protein; RBC, red blood cell; IQR, interquartile range

### Pathological correlates of renal Toll-like receptor 4 expression

**Fig. 3:**
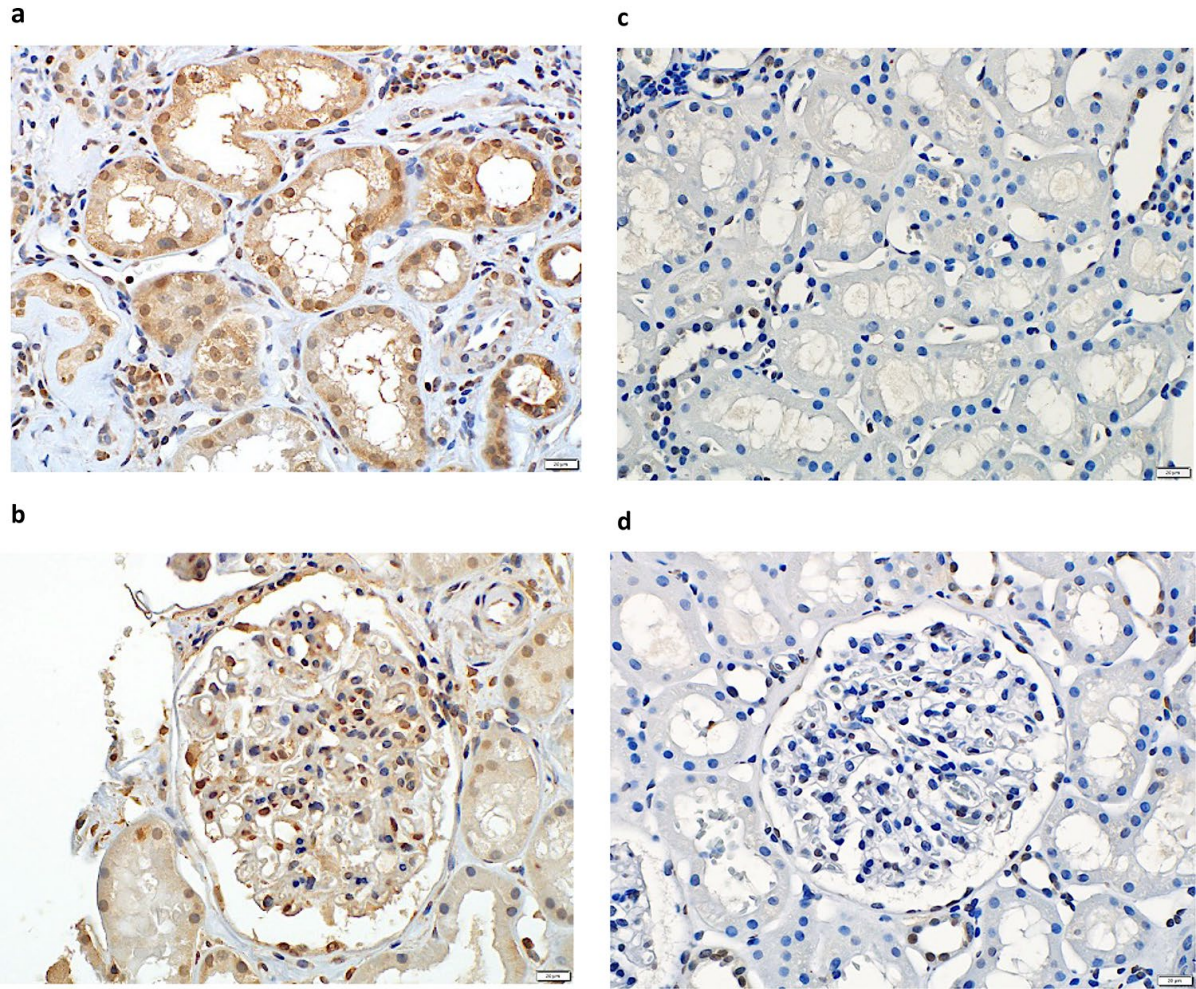
TLR4 expression in DN renal tissues. **A** Positive TLR4 expression in the proximal tubules of patients with DN. **B** Positive TLR4 expression in the glomerular epithelial cells from patients with DN. **C** Negative TLR4 expression in the proximal tubules of patients with thin basement membrane disease. **D** Negative TLR4 expression in the glomerular epithelial cells of patients with thin basement membrane disease. (Original magnification, × 400). Abbreviations: TLR, Toll-like receptor; DN, diabetic nephropathy

Representative TLR4 immunostaining patterns are shown in Fig. 3. Glomerular TLR4 positivity was observed in 59% of the specimens, and moderate-to-severe proximal tubular TLR4 expression was observed in 47% of the specimens. There was a significant difference in the severity of glomerular lesions across the levels of glomerular epithelial TLR4 expression (*p* = 0.015). The extent of IFTA and interstitial inflammatory infiltration differed significantly among the levels of proximal tubular TLR4 expression (*p* = 0.043 and *p* = 0.012, respectively) (Table 2). In the multivariable logistic regression model adjusted for relevant covariates, none of the histological factors showed a statistically significant independent association with the TLR4 expression (Supplementary Table 1). No significant differences in sex, age, BMI, BP, eGFR, HbA1c, total cholesterol levels, or proteinuria were observed across the different levels of TLR4 expression (glomerular or tubular) (Supplementary Table 2).

**Table 2:** Comparison of histological severity of DN according to TLR4 expression

TLR4 expression in glomerular epithelial cells	*p*-value
Glomerular class	0.015
IFTA	0.060
Interstitial inflammation	0.430
Arteriosclerosis	0.950
Arteriolar hyalinosis	0.305

TLR4 expression in the proximal tubule	*p*-value
Glomerular class	0.248
IFTA	0.043
Interstitial inflammation	0.012
Arteriosclerosis	0.275
Arteriolar hyalinosis	0.215

TLR, Toll-like receptor; DN, diabetic nephropathy; IFTA, interstitial fibrosis and tubular atrophy

### Renal outcome correlates with renal Toll-like receptor 4 expression

**Fig. 4:**
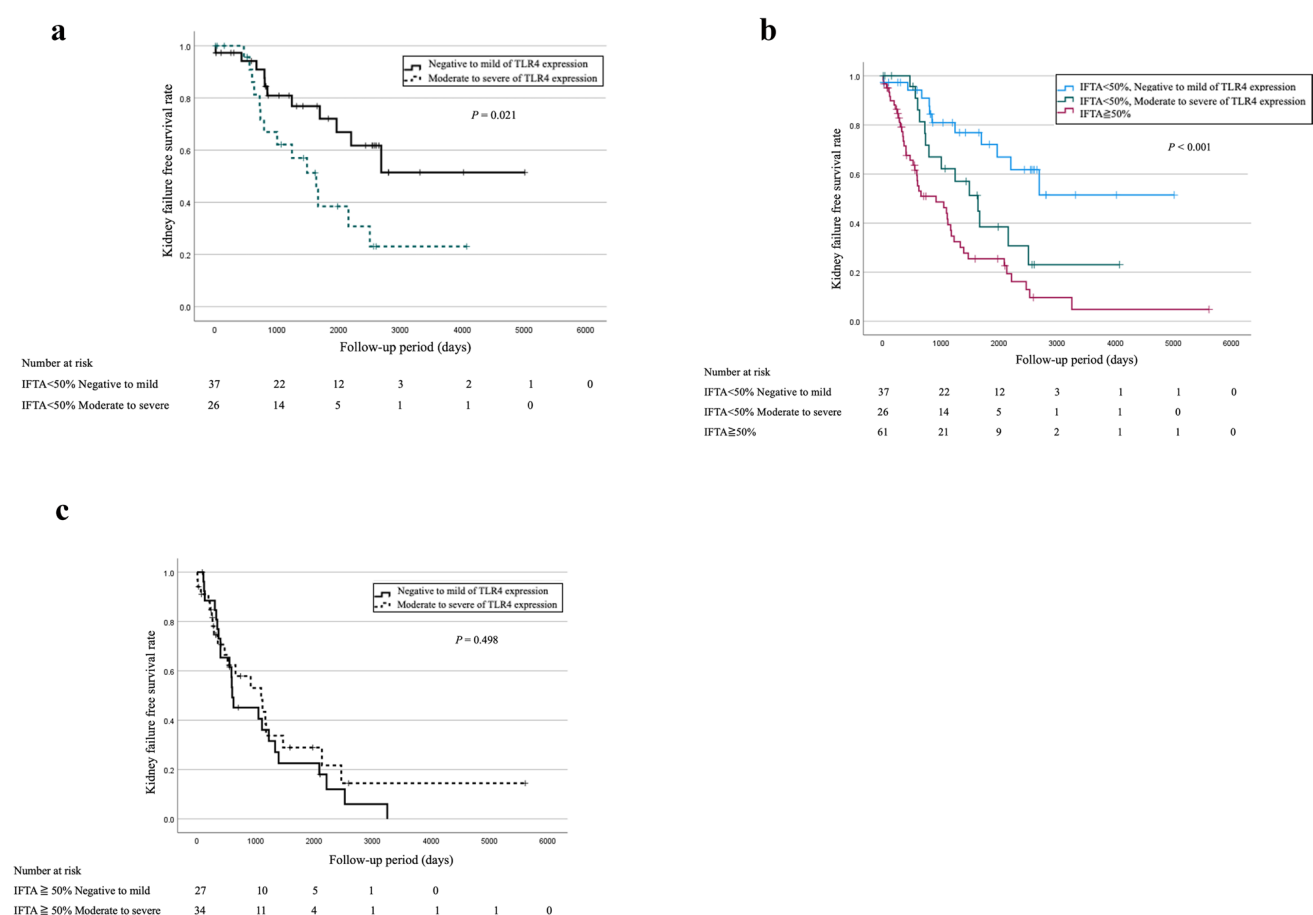
Kidney failure-free survival rates in patients with DN. **A** Patients with IFTA < 50%: Compared with the group with negative-to-mild positive TLR4 expression and moderate-to-severe positive TLR4 expression in the proximal tubules. **B** Patients with IFTA ≥ 50%: Compared with the group with negative-to-mild positive TLR4 expression and moderate-to-severe positive TLR4 expression in the proximal tubules. **C** Compared with patients with IFTA < 50% and negative-to-mild positive TLR4 expression and IFTA < 50% and moderate-to-severe positive TLR4 expression and all patients with IFTA ≥ 50%. Abbreviations: DN, diabetic nephropathy; TLR, Toll-like receptor; IFTA, interstitial fibrosis and tubular atrophy

In the overall cohort, follow-up data on prognosis were available for 124 patients, with a median follow-up of 2.2 years. TLR4 expression was not significantly associated with the kidney outcomes. However, among patients with IFTA involving < 50% of the cortical area (n = 63), those with moderate-to-severe proximal tubular TLR4 expression had significantly poorer kidney failure-free survival than those with negative-to-mild expression (*p* = 0.021) (Fig. 4). Regardless of the TLR4 status, patients with IFTA ≥ 50% had the worst renal prognosis (Fig. 4). We evaluated the interaction between TLR4 and IFTA category by including an interaction term (TLR4 × IFTA) in the Cox model; the P-value for interaction was 0.638. Glomerular TLR4 expression was not significantly associated with renal outcomes (Supplementary Fig. 2).

### Clinical characteristics of the low‑interstitial fibrosis and tubular atrophy subgroup

The patients were stratified according to the extent of IFTA and level of TLR4 expression, and their baseline characteristics are summarised in Table 3. Within the subgroup with IFTA < 50%, patients with negative-to-mild tubular TLR4 expression had lower systolic BP, less proteinuria, and lower total cholesterol than the patients with moderate-to-severe expression (median, 138 vs. 155 mmHg [*p* = 0.029]; 3.36 g/gCr vs. 5.8 g/gCr [*p* = 0.005]; and 192 mg/dL vs. 227 mg/dL [*p* = 0.006], respectively), whereas the eGFR and CRP did not significantly differ between the groups.

**Table 3:** Comparison of clinical characteristics among the three DN histological groups

	IFTA < 50% Negative-to-mild TLR4 expression n = 37	IFTA < 50% Moderate-to-severe TLR4 expression n = 26	IFTA ≥ 50% Negative-to-severe TLR4 expression n = 61	*p*-value
Sex (n, male/female)	22/15	19/7	49/12	0.08^*^
Age, years, median, [IQR]	62 (46–70)	62 (48.8–71)	58 (49–66)	0.684^**^
BMI, kg/m^2^, median, [IQR]	26.0 (21.0–30.1)	26.8 (24.1–29.1)	24.1 (23.1–27.9)	0.189^**^
Systolic BP, mmHg, median, [IQR]	138 (123–156)	155 (133–166)^a^	147 (132–165)^a^	0.047^**^
Diastolic BP, mmHg, median, [IQR]	79 (69–86)	81 (78–92)	80 (70–91)	0.336^**^
Antihypertensive drugs, n, (yes/no)	29/8	20/6	55/6	0.170^*^
eGFR, mL/min/1.73 m^2^, median, [IQR]	52.1 (39.5–65.3)	48.8 (37.1–59.8)	25.7 (18.4–35.1)^a,b^	< 0.001^**^
HbA1c, %, median, [IQR]	6.7 (6.2–7.7)	6.8 (6.2–8.4)	6.4 (5.8–7.2)	0.124^**^
Serum albumin, mg/dL, median, [IQR]	3.4 (2.9–4.1)	2.9 (1.8–3.8)^a^	2.8 (2.3–3.3)^a^	< 0.001^**^
Total cholesterol, mg/dL, median, [IQR]	192 (159–222)	227 (195–282)^a^	202 (168–234)	0.022^**^
CRP, mg/dL, median, [IQR]	0.09 (0.03–0.11)	0.1 (0.05–0.6)	0.11 (0.06–0.6)^a^	0.026^**^
Proteinuria, g/gCr, median, [IQR]	3.4 (1.5–4.7)	5.8 (2.9–14.4)^a^	6.3 (3.9–10.4)^a^	0.002^**^
Haematuria (RBC ≥ 5/HPF), n, (yes/no)	24/13	10/16	30/31	0.103^*^
Diabetic retinopathy, n, (yes/no)	18/11	13/5	31/21	0.970^*^

TLR, Toll-like receptor; DN, diabetic nephropathy; IFTA, interstitial fibrosis and tubular atrophy; BMI, body mass index; BP, blood pressure; eGFR, estimated glomerular filtration rate; CRP, C-reactive protein; RBC, red blood cell; IQR, interquartile range

^a^*p* < 0.05 vs. IFTA < 50%, negative-to-mild TLR4 expression

^b^*p* < 0.05 vs. IFTA < 50%, moderate-to-severe TLR4 expression

^*^Pearson’s chi-squared test, ^**^Kruskal–Wallis test

## Discussion

In this study, we hypothesised that TLR4 expression reflects the severity of renal histopathological injury in human DN and is associated with renal outcomes. Consistent with this hypothesis, we observed significant differences in histopathological damage across different TLR4 expression levels. However, with respect to renal outcomes, the prognostic impact of TLR4 was not uniform across fibrosis strata: in the stratified analyses, higher TLR4 expression was associated with a higher risk of kidney failure in patients with less fibrosis (IFTA < 50%), but this association was not evident in those with advanced fibrosis (IFTA ≥ 50%). Notably, the formal interaction between TLR4 and fibrosis category was not statistically significant; thus, these findings should be interpreted as stratified associations rather than definitive effect modification.

The novelty of this study lies in the histopathological analysis of 146 patients with DN and integration of long-term prognostic follow-up data.

The difference in interstitial inflammation across different levels of TLR4 expression was remarkable. Mudaliar et al. showed that exposing human proximal tubular epithelial cells to hyperglycaemia increased TLR2/TLR4 expression, HMGB1 (an endogenous ligand of TLR4) release, and NF-κB activation, along with concomitant increases in inflammatory cytokines [7]. Lin et al. reported that TLR4 was highly expressed in the renal tubules of patients with diabetic nephropathy and that its staining intensity positively correlated with tubular macrophage infiltration and HbA1c levels [6]. Furthermore, HMGB1 was highly expressed in the tubular compartment. In this study, significant differences in tubulointerstitial inflammatory cell infiltration were observed across different levels of TLR4 expression, suggesting the possible involvement of TLR4 in progressive histological injury. However, no significant differences were observed in clinical parameters, such as HbA1c, across the levels of TLR4 expression.

TLR4 is activated by endogenous ligands upregulated in DNs, such as HMGB1, HSP, and fibronectin [20]. The expression of these endogenous ligands is upregulated under conditions of hyperglycaemia, proteinuria, and dyslipidaemia [21–23]; notably, proteinuria induces HSP70 more potently than hyperglycaemia [22]. In patients with a relatively preserved tubulointerstitial compartment (IFTA < 50%), elevated tubular TLR4 expression could reflect a secondary response to the production of endogenous TLR4 ligands triggered by proteinuria and dyslipidaemia.

There was no association between TLR4 expression and kidney failure-free survival in any patient; however, in the group of patients with < 50% IFTA, moderate-to-severe TLR4 expression in the renal tubules was associated with poor renal prognosis. In patients with microalbuminuria, glomerular TLR4 expression was associated with subsequent loss of kidney function [8]. These results suggest that TLR4 contributes to disease progression in the early stages of DN. In this study, patients with IFTA < 50% and those with moderate-to-severe tubular TLR4 expression had higher systolic BP, greater proteinuria, and elevated total cholesterol levels than those in the negative-to-mild group. In patients with DN and less advanced IFTA (e.g. IFTA < 50%), characterised by high TLR4 expression, rigorous control of these modifiable factors is imperative. Notably, no association was detected between clinical parameters and TLR4 expression in the overall cohort, underscoring the importance of assessing TLR4 expression by immunostaining renal tissue.

Preclinical studies have consistently demonstrated that targeting TLR4 via genetic deletion or pharmacological antagonism attenuates DN progression. In diabetic mice, the TLR4 antagonist CRX-526 reduced albuminuria independent of changes in blood glucose or systolic BP, ameliorated glomerulosclerosis and tubulointerstitial injury, and suppressed the expression of chemokine ligand (CCL) 2, osteopontin, and CCL5, with consequent reductions in macrophage infiltration and collagen deposition [24]. Mice with a podocyte-specific deletion of TLR4 exhibit reversal of foot process effacement and marked improvement in proteinuria [25]. Furthermore, natural compounds, such as curcumin, paclitaxel, and berberine, ameliorate DN by modulating the TLR4 signalling pathway [26].

This study has some limitations. First, a key limitation is the potential selection bias inherent to biopsy-based DN cohorts. In routine clinical practice, kidney biopsies are primarily performed in patients with diabetes when the clinical course is atypical—for instance, in the absence of diabetic retinopathy, the presence of hematuria, an acute exacerbation of proteinuria, or a rapid decline in kidney function. Consequently, our cohort may over-represent atypical or more severe clinical presentations and may not fully reflect the population of patients with DN managed without biopsy. This may limit the generalizability of the observed findings regarding TLR4 expression to unselected or earlier-stage DN populations. Second, TLR4 expression was evaluated using a semiquantitative measurement. Third, DN treatment patterns evolved between 2004 and 2021. Since data on RAS inhibitors, statins, and SGLT2 inhibitors were not systematically recorded, we could not adjust for medication exposure. These therapies significantly impact clinical parameters and might influence our results. Consequently, this cross-sectional study is hypothesis-generating and does not establish causality.

## Conclusion

In conclusion, TLR4 expression may be involved in histological injury and adverse renal outcomes in DN, particularly in patients with limited interstitial fibrosis (IFTA < 50%). Together with prior mechanistic and preclinical evidence, our findings support the contributory role of TLR4 signalling in DN progression before extensive interstitial fibrosis develops and highlight TLR4 and its endogenous ligands as potential therapeutic targets. Rigorous control of modifiable factors associated with TLR4 activation (e.g. proteinuria and dyslipidaemia) may be especially relevant in patients with DN with high tubular TLR4 expression.

## Supplementary Information

Below is the link to the electronic supplementary material.

Supplementary file1 (DOCX 285 KB)

Supplementary file2 (DOCX 345 KB)

Supplementary file3 (DOCX 15 KB)

Supplementary file4 (DOCX 15 KB)

## Data Availability

The datasets underlying this article are available from the corresponding author upon reasonable request.
