# Supplementary material for: Progression of diabetic nephropathy and adverse renal outcomes: possible involvement of Toll-like receptor 4 expression
Source: Clin Exp Nephrol. 2026 Mar 24;30(6):866–74. doi: 10.1007/s10157-026-02849-2 (PMC13242377; doi:10.1007/s10157-026-02849-2)
Supplement: Supplementary file 1 — Supplementary file1 (DOCX 285 KB) [file 10157_2026_2849_MOESM1_ESM.docx]

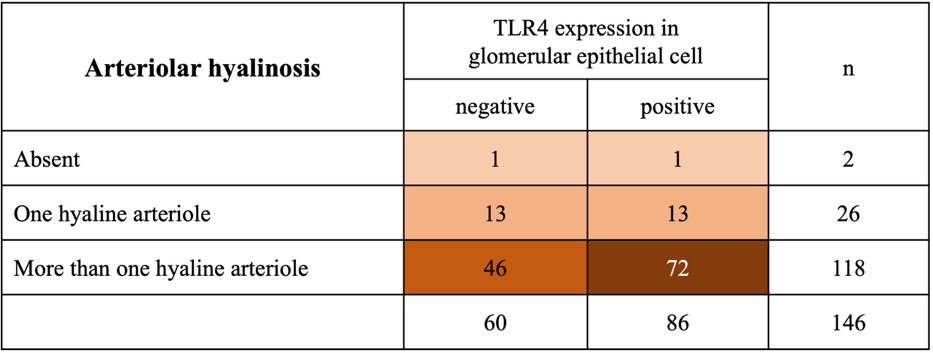


**
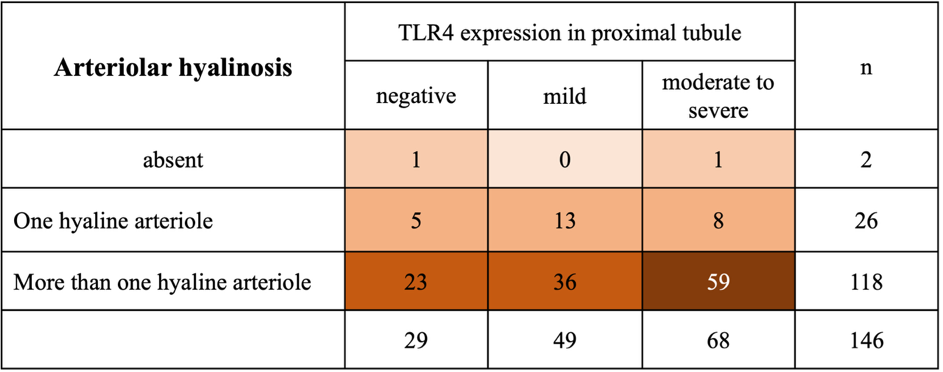
**


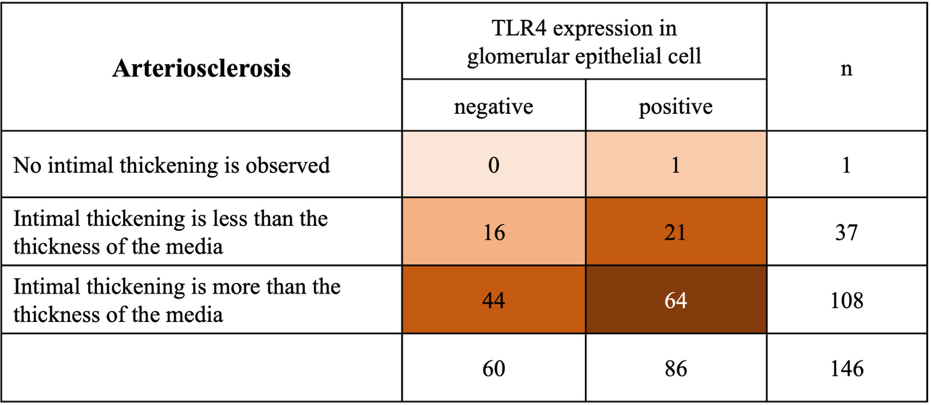


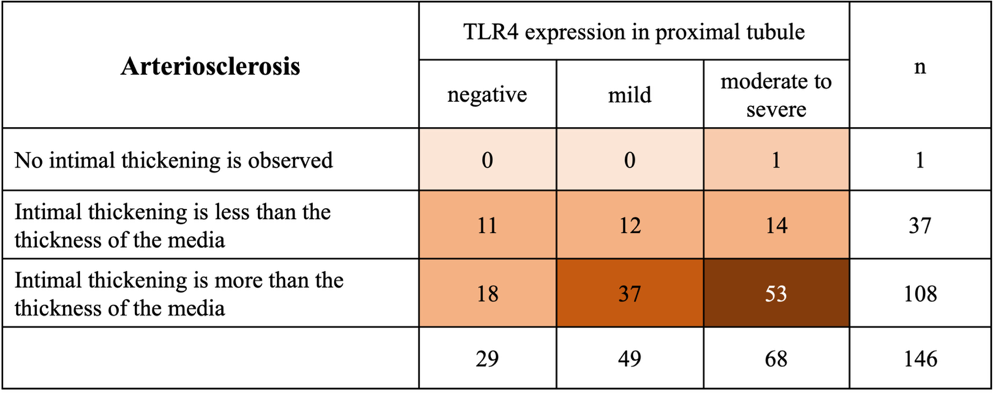


**Supplementary Figure 1**

**Supplementary Fig. 1**

Relationship between TLR4 expression and the histological features of DN.

TLR, Toll-like receptor; DN, diabetic nephropathy; IFTA, interstitial fibrosis and tubular atrophy; LM, light microscopy
