# Supplementary material for: Progression of diabetic nephropathy and adverse renal outcomes: possible involvement of Toll-like receptor 4 expression
Source: Clin Exp Nephrol. 2026 Mar 24;30(6):866–74. doi: 10.1007/s10157-026-02849-2 (PMC13242377; doi:10.1007/s10157-026-02849-2)
Supplement: Supplementary file 2 — Supplementary file2 (DOCX 345 KB) [file 10157_2026_2849_MOESM2_ESM.docx]

**a**

**
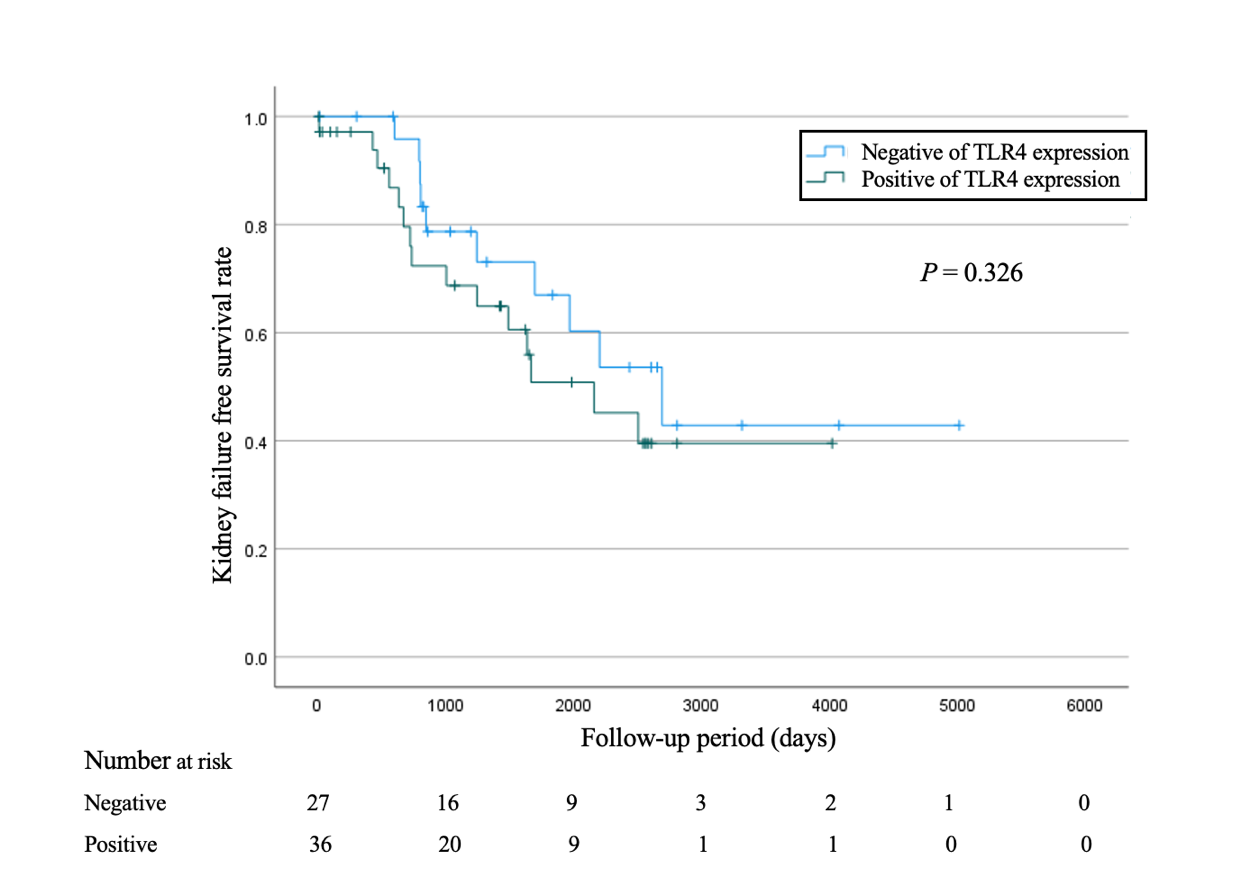
**

**b**

**
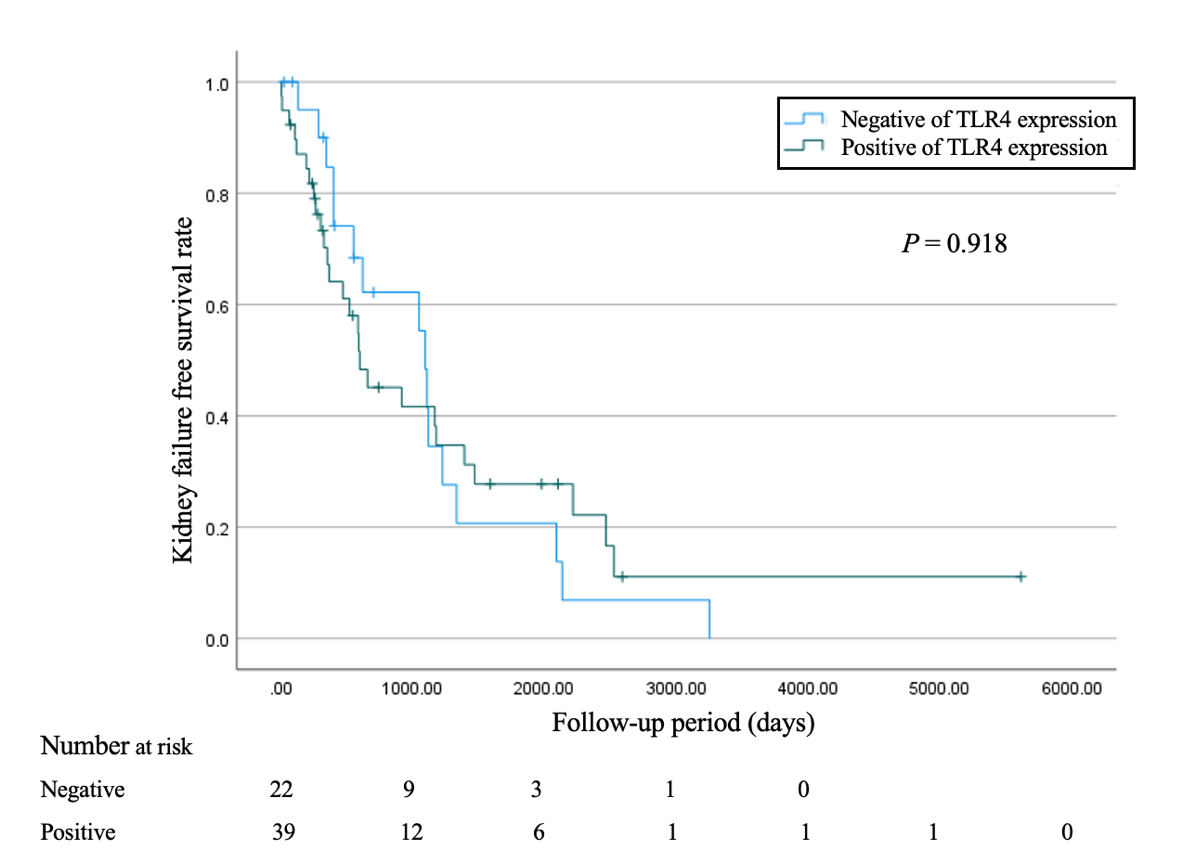
**

**c**

**
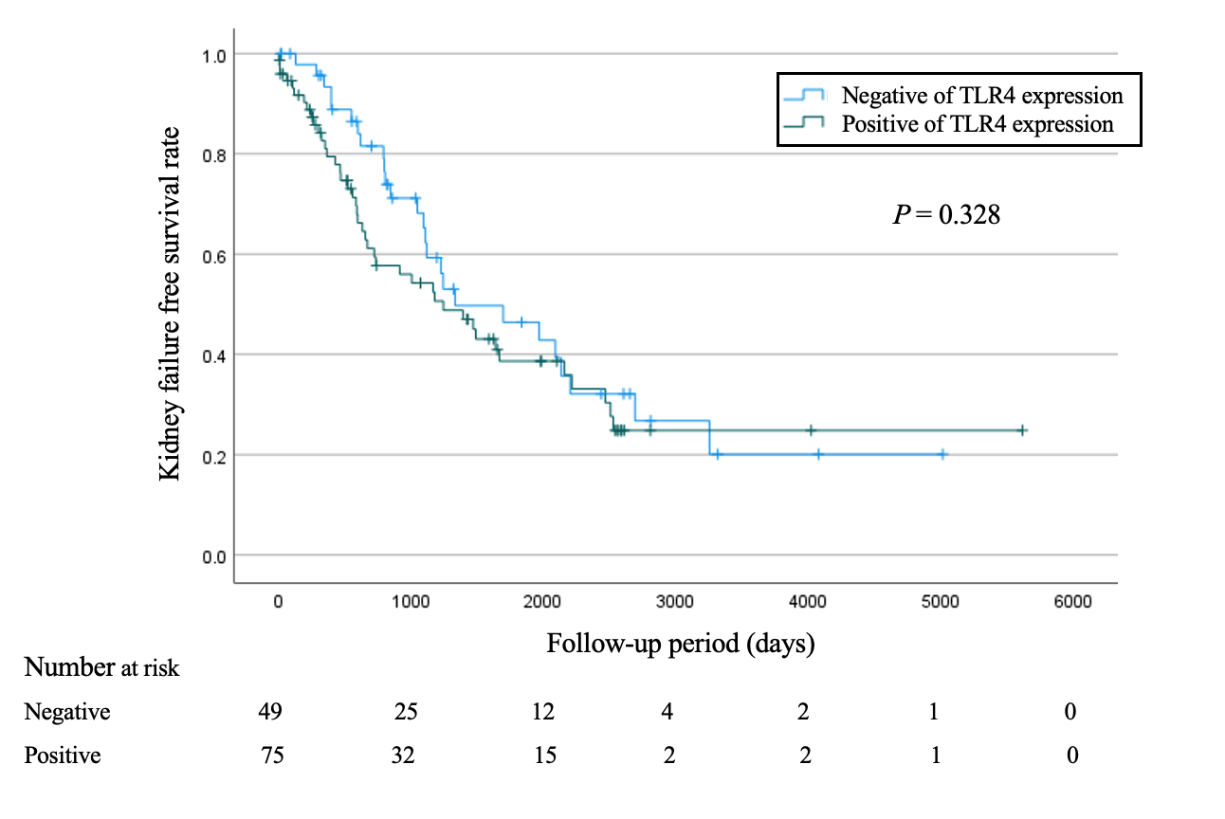
**

**Supplementary Figure 2**

**Supplementary Fig. 2** Kidney failure-free survival rates in patients with DN

(A) Patients with IFTA < 50%: Compared with the group with negative and positive TLR4 expression in the glomeruli.

(B) Patients with IFTA ≥ 50%: Compared with the group with negative and positive TLR4 expression in the glomeruli.

(C) Comparison of kidney failure-free survival between patients with negative and positive glomerular TLR4 expression in the entire cohort.

DN, diabetic nephropathy; TLR, Toll-like receptor; IFTA, interstitial fibrosis and tubular atrophy
