# Supplementary material for: Progression of diabetic nephropathy and adverse renal outcomes: possible involvement of Toll-like receptor 4 expression
Source: Clin Exp Nephrol. 2026 Mar 24;30(6):866–74. doi: 10.1007/s10157-026-02849-2 (PMC13242377; doi:10.1007/s10157-026-02849-2)
Supplement: Supplementary file 3 — Supplementary file3 (DOCX 15 KB) [file 10157_2026_2849_MOESM3_ESM.docx]

|  | Variables associated with  glomerular TLR4 expression | | Variables associated with  tubular TLR4 expression | |
| --- | --- | --- | --- | --- |
|  | Odds Ratio [95% CI] | *p*-value | Odds Ratio [95% CI] | *p*-value |
| Glomerular class | 1.39 [0.96 to 2.02] | 0.09 | 0.97 [0.69 to 1.36] | 0.84 |
| IFTA | 1.23 [0.77 to 1.97] | 0.39 | 1.40 [0.90 to 2.15] | 0.13 |
| Interstitial inflammation | 0.99 [0.51 to 1.92] | 0.97 | 1.50 [0.81 to 2.76] | 0.20 |
| Arteriosclerosis | 0.74 [0.33 to 1.65] | 0.46 | 1.23 [0.60 to 2.54] | 0.57 |
| Arteriolar hyalinosis | 1.01 [0.42 to 2.44] | 0.98 | 1.11 [0.50 to 2.48] | 0.80 |

**Supplementary Table 1.** Association between histological characteristics and TLR4 expression

TLR, Toll-like receptor; IFTA, interstitial fibrosis and tubular atrophy; CI, confidence interval.
