# Supplementary material for: Progression of diabetic nephropathy and adverse renal outcomes: possible involvement of Toll-like receptor 4 expression
Source: Clin Exp Nephrol. 2026 Mar 24;30(6):866–74. doi: 10.1007/s10157-026-02849-2 (PMC13242377; doi:10.1007/s10157-026-02849-2)
Supplement: Supplementary file 4 — Supplementary file4 (DOCX 15 KB) [file 10157_2026_2849_MOESM4_ESM.docx]

**Supplementary Table 2.** Correlation of clinical characteristics with different TLR4 expression levels

|  | Association with TLR4 expression in glomerular epithelial cells | Association with TLR4  expression in proximal tubular cells |
| --- | --- | --- |
|  | *p*-value | *p*-value |
| Sex | 0.94 | 0.63 |
| Age | 0.91 | 0.82 |
| BMI | 0.95 | 0.74 |
| SBP | 0.08 | 0.30 |
| DBP | 0.24 | 0.65 |
| eGFR | 0.90 | 0.27 |
| HbA1c | 0.45 | 0.57 |
| Total cholesterol | 0.72 | 0.38 |
| proteinuria | 0.08 | 0.06 |

TLR, Toll-like receptor; DKD, diabetic kidney disease; IFTA, interstitial fibrosis and tubular atrophy; BMI, body mass index; SBP, systolic blood pressure; DBP, diastolic blood pressure; eGFR, estimated glomerular filtration rate.
